# COVID-19 pandemic: implementing control measures in Africa using the 'SHEF2' model

**DOI:** 10.11604/pamj.supp.2020.37.1.24057

**Published:** 2020-09-03

**Authors:** Frankline Sevidzem Wirsiy, Claude Ngwayu Nkfusai, Denis Ebot Ako-Arrey, Eugene Vernyuy Yeika, Florence Titu Manjong, Dongmo Kenfack Esther, Rosette Boseme Nzoyom, Catherine Atuhaire, Jean-Claude Kindzeka Wirsiy, Samuel Nambile Cumber

**Affiliations:** 1 United Nations Office for the Coordination of Humanitarian Affairs, COVID-19 Response, Yaoundé, Cameroon,; 2 United Nations Volunteer, COVID-19 Response, Bafoussam, Cameroon,; 3 Department of Public Health and Hygiene, Faculty of Health Sciences, University of Buea, Buea, Cameroon,; 4 Saint Louis University Institute of Health and Biomedical Sciences, Douala, Cameroon,; 5 Georgetown University's Center for Global Health Practice and Impact (CGHPI), TIDE-Cameroon Program, Yaounde, Cameroon,; 6 Department of Public Health, School of Nursing and Public Health, University of Kwa-Zulu Natal, Durban, South Africa,; 7 United Nations Migration Agency, International Organization for Migration, Kinshasha Democratic Republic of Congo,; 8 Department of Public Health, Institute of Tropical Medicine, Antwerp, Belgium,; 9 Department of Nursing, Grand Canyon University, Arizona United State of America,; 10 Faculty of Medicine, Department of Nursing, Mbarara University of Science and Technology, Uganda,; 11 Centre for Health Systems Research and Development, University of the Free State, Bloemfontein, South Africa,; 12 School of Health Systems and Public Health, Faculty of Health Sciences, University of Pretoria, Pretoria, South Africa,; 13 Faculty of Health Sciences, University of the Free State, Bloemfontein, South Africa,; 14 Institute of Health and Care Sciences, The Sahlgrenska Academy, University of Gothenburg, Gothenburg, Sweden

**Keywords:** SHEF2 model, COVID-19, pandemic, control measures, Africa

## Abstract

The COVID-19 Pandemic in Africa is a severe reminder of the brunt of emerging and re-emerging infectious pathogens and the need for simple, context-oriented, and sustainable health models to combat them when the need arises. In this commentary, an analytical discursive approach was chosen to owe to Africa's unique situation of weak health systems, with most of its member states showing an initial reluctance to deal openly with the COVID-19 situation. This paper discusses five major control measures doped the SHEF2 Model i.e. ("SHEF2"- S: Social distancing, H: Hands, E: Elbows, F: Face, F: Feel) of COVID-19 implemented in Africa. We also review the issues related to implementing SHEF2 control measures in Africa. The measures being taken in Asia, Europe, and North America such as social distancing and regular hand washing are a particular challenge for African countries with dense populations, unequal access to water, and limited social safety nets. COVID-19 is challenging the public health and socio-political systems of all affected African countries. The burden of COVID-19 demands rapid and decisive action to be taken, yet the comparison shows how difficult it is was for an unknown new coronavirus disease. In line with the steps being taken across the globe to control and contain COVID-19 pandemic, African countries are preparing for the great effects of this pandemic and ensuing deep recession thus the reason we assert, the greater hope for African countries is implementing an aggressive SHEF2 model strategy. The spread of the pandemic will eventually stop, and the international system will find a balance, but most of the damage will be felt particularly by Africa.

## Commentary

Coronavirus disease 2019 (COVID-19) spreading in African countries with weak health systems is a call for concern. With governments in Asia, Europe, and the United States fight despairing national combats of their own against COVID-19, the African continent may seem a remote concern. Also, the COVID-19 Pandemic is a severe reminder of the brunt of emerging and re-emerging infectious pathogens and the need for constant surveillance, prompt diagnosis, and robust research [1] to understand the basic biology of new organisms and human susceptibilities to them, as well as to develop effective control countermeasures. COVID-19 was confirmed to have spread to Africa on 14 February 2020 and this first confirmed case was in Egypt [2] such that exposure surely occurred earlier. Most other cases since then were imported from Europe; fewer came from the Americas and Asia [3]. In responding to the COVID-19 Pandemic, Africa needs to adhere to the SHEF2 model (“SHEF2”- S: Social distancing, H: Hands, E: Elbows, F: Face, F: Feel) control measures. Thus, discussing and implementing these measures, involving all stakeholders is therefore of prime importance. In this paper, we present an analytical discursive approach owing to Africa´s unique situation of weak health systems, with most of its member states showing an initial reluctance to deal openly with the COVID-19 situation. Commenting on issues related to implementing SHEF2 model control measures in an ‘African Perspective´ is critical. Equally, highlighting these control features of Africa in controlling COVID-19 and the lessons learned could help policymakers to modify outbreak management strategies for better preparedness and earlier response in the future.

**Implementing the SHEF2 Model to Control Coronavirus (“SHEF”) in Africa:** a summary of the key features of the SHEF2 model to control coronavirus in Africa is shown in Table 1.

**Table 1:** summary of keys features of the SHEF2 model against the COVID-19 pandemic

SHEF2 Model	Keys features
**S: Social Distancing**	- By maintaining such social distancing, you are helping to avoid breathing in any droplets from someone who sneezes or coughs nearby
	- Social distancing delays and reduces the magnitude of outbreaks of pandemic influenzas including COVID-19
	- At the individual level, social distancing involves the use of non-contact greetings, maintaining at least one meter distance between yourself and other people, and staying home when ill with flu-like symptoms.
	- At the community level, social distancing involves closure of any events or settings in which people gather together, including schools, workplaces, houses of worship, and cultural, social and sports events
**H: Hands**	- Our hands are the front lines in the war against Covid-19
	- Hands represent one of the most common ways that COVID-19 spreads from one person to the next and as such,
	- The cheapest, easiest, and most important ways to prevent the spread of a virus is to wash your hands frequently with soap and running water. It is simple, but it is very pertinent
**E: Elbows and disposable tissues**	- Covering your nose and mouth when you cough or sneeze (using a disposable tissue, clothing or your elbow), limits the spread of coronavirus.
	- While wearing a mask can help limit the spread of some respiratory diseases, it is not enough on its own and handwashing and respiratory hygiene are critically important
	- People who cough/sneeze into a disposable tissue are recommended not to forget to throw the tissue into a closed bin and wash their hands straight afterwards
**F: Face (Avoiding to touch the face)**	- Avoiding to touch the face, particularly the eyes, nose or mouth has been reported to prevent coronavirus from entering your body
	- In Africa, one of the more difficult challenges in public health has been to teach people to stop touching the facial mucous membranes-the eyes, nose and mouth, all entry portals for the SARS COV-2.
	- If you never touch your facial mucous membranes, you're less likely to be sick again from any viral respiratory infection
**F: Feel (Know your symptoms)**	- If you have a fever, cough and difficulty breathing, seek medical attention and call in advance.
	- The most common symptoms of COVID-19 are fever, tiredness, and dry cough even though some patients may have aches and pains, nasal congestion, runny nose, sore throat or diarrhea.
	- These symptoms are usually mild and begin gradually with some people becoming infected but asymptomatic and don't feel unwell

**S: Social Distancing:** social distancing is an accepted strategy that has been implemented in the world including Africa to delay and reduce the magnitude of outbreaks of pandemic influenza [4,5]. In this era of COVID-19, social distancing is necessary at the individual and community levels, because following the four stages of transmission of COVID-19, transmission occurs frequently from person-to-person and infection causes severe illness in up to 20 percent of people [5]. By maintaining such social distancing, you are helping to avoid breathing in any droplets from someone who sneezes or coughs close. At the individual level, social distancing involves the use of non-contact greetings, maintaining at least one-meter distance between yourself and other people, and staying home when ill with flu-like symptoms [3]. At the community level, social distancing involves closure of any events or settings in which people gather together, including schools, workplaces, houses of worship, and cultural, social, and sports events [6]. In the African context, coupled with high poverty levels, crowded cities, and vast slums; social distancing is problematic to implement. According to estimates by UN-Habitat, 200 million people in sub-Saharan Africa were living in slums in 2010, the highest rate in the world [7]. North Africa had another 12 million slum dwellers; that was just 13.3 percent of its urban residents, the lowest rate in the developing world [7]. Social distancing a distant dream in Africa´s slums. On a continent where two or three generations often live under the same roof, and personal space is lacking, it may be more difficult to impose social distancing to slow down the coronavirus spread in Africa compared to Europe, Asia, and the Americas. “Spending time together is the norm,” in the African context says Gilles Yabi, founder of the West African think tank Wathi, of a culture where family gatherings and big crowds go hand in hand [8]. Social distancing is a containment solution that assumes certain things that are just not true of crowded cities like Lagos and many such cities across Africa. For one, people have to be able to embrace reduced or zero productivity for an extended period, without that leading to immediate disastrous consequences. In Africa, the majority of people live on incomes earned largely daily. As such, this represents millions of families who can only start buying or making meals when the primary breadwinner closes from work on any given day. For such people, the possibility of catching a previously unheard-of illness is a far less dangerous one than the knowledge that not having anything to eat which becomes always a sunrise away [9].

**H: Hands:** COVID-19 spread when mucus or droplets containing the virus get into the body through the eyes, nose, or throat. Most often, this happens through the hands. Thus the Hands represent one of the most common ways that the virus spreads from one person to the next and as such, our hands are the front lines in the war against Covid-19. The Centers for Disease Control and Prevention (CDC) recommends washing hands with soap and running water as the top way to clean our hands [3]. “But if soap and water are not available, using a hand sanitizer with at least 60% alcohol can help,” the CDC says [3]. During a global pandemic, one of the cheapest, easiest, and most important ways to prevent the spread of a virus is to wash your hands frequently with soap and running water. It is simple, but it is very important. In Africa, the lack of adequate sanitation, potable water aggravates the spread of diseases like COVID-19 and avoidable deaths. The COVID-19 pandemic presents a particular challenge for the water, hygiene, and sanitation (WASH) sector. People living in densely populated settings-including urban areas, refugee camps, internally displaced people settlements, and prisons-are especially vulnerable in many of these places, as they rely on community facilities, such as shared water points and communal toilets, and are dependent on private vendors and tanker trucks rather than water piped in from a water utility [10]. Even when there is access to water, it is often intermittent and only available a few hours per day. In many places in Africa, there is also limited access to soap. There is now a need for WASH activities to be considered an essential public health intervention.

**E: Elbows and disposable tissues:** droplets spread the coronavirus [5]. By following respiratory hygiene, you protect the people around you from contracting COVID-19. The Severe Acute Respiratory Syndrome -Coronavirus 2 (SARS COV-2) spreads through droplets in the air from coughing and sneezing. This means that by covering your nose and mouth when you cough or sneeze (using a disposable tissue, clothing, or elbow), you can limit the spread of the virus [5]. While wearing a mask can help limit the spread of some respiratory diseases, it is not enough on its own, and handwashing and respiratory hygiene are critically important [3]. The trend in African countries is such the more people wear mostly handmade face masks, the lesser they tend to wash their hands regularly and practice hygiene measures. The World Health Organization (WHO) recommends using masks if you are coughing and sneezing, have a mild suspected case of COVID-19 or you are caring for someone with suspected COVID-19 [5]. People who cough/sneeze into a disposable tissue are recommended not to forget to throw the tissue into a closed bin and wash their hands straight afterward. Thus this should be a habit in the lifestyle of Africans during COVID-19 Pandemic.

**F: Face (Avoiding to touch the face):** avoiding to touch the face, particularly the eyes, nose or mouth has been reported to prevent the virus from entering your body [3]. It has been shown scientifically that, on average, people touch their faces 23 times an hour [5]. We know fetuses touch their faces in utero, which means we all got into the habit before we were even born. Hands touch too many surfaces and can quickly pick up SARS COV-2. Once contaminated, hands can transfer the virus to your face, from where the virus can move inside your body, making you feel unwell. In Africa, same as other parts of the world, one of the more difficult challenges in public health has been to teach people to wash their hands frequently as well as to stop touching the facial mucous membranes-the eyes, nose, and mouth, all entry portals for the SARS COV-2. We concur with the advice of sending a message such as ‘do not touch them!´ - If you never touch your facial mucous membranes, you´re less likely to be sick again from any viral respiratory infection [5].

**F: Feel (Know your symptoms):** keeping informed as local health authorities provide the latest information on the situation in individual African countries should be the way out. Following the specific instructions of WHO measures in addition to government measures and calling in advance in case someone is experiencing symptoms will allow them to direct you to the appropriate health facility. This serves to protect the population and to help prevent the spread of COVID-19. If you have a fever, cough, and difficulty breathing, seek medical attention, and call in advance. The most common symptoms of COVID-19 are fever, tiredness, and dry cough [5] even though some patients may have aches and pains, nasal congestion, runny nose, sore throat, or diarrhea. These symptoms are usually mild and begin gradually with some people becoming infected but asymptomatic and don't feel unwell [3]. Most people (about 80%) recover from the disease without needing special treatment. The message that has been running across most African countries is “If you feel unwell, stay home.” Initially, Africans thought COVID-19 was a “Whiteman illness” and there were rumors about COVID-19 not able to infect Africans. The issue of staying at home is difficult in the African context due to socio-economic factors. This notwithstanding, WHO has reiterated that if an individual is feeling/experiencing the signs and symptoms of COVID-19, they should “Please follow all instructions provided by your local health authorities.” Africans suffer from indiscipline such that some of them even run away from quarantine centers as is the case of Cameroon. When highlighting Africa´s response to that of other COVID-19 affected regions, it should be kept in mind that Africa is unfavorable in many areas, from basic hygiene needs to health infrastructure. Other pertinent factors are the presence of people living in unfavorable conditions in major cities, as well as communities of people displaced in sub-Saharan African countries due to socio-political crisis and forced to migrate to other countries as well as live in non-sterile environments in camps. Tackling cases in rural areas of Africa that often lack the resources of urban centers will pose an immense challenge for already strained health systems. The key factor is the continent's recent experience with viruses such as Ebola and Lassa fever. This experience warrants the provision of significant infrastructure support for dealing with outbreaks, but insufficient health infrastructure and problems in accessing clean water across much of the continent will still raise concerns. The international community needed to act urgently to overcome the chronic problems that will hamper the fight against the COVID-19 pandemic in African countries. The probable fact is that African countries need support in managing the health crisis, and preparing for durable economic fallouts.

In conclusion, implementing the SHEF2 model i.e. (“SHEF2”-Social distancing, Hands, Elbows, Face, Feel), are the key control measures against COVID-19 in the African continent. It is worthy to reiterate that, the measures being taken in Asia, Europe, and North America such as physical (social) distancing and regular hand washing are a particular challenge for African countries with limited internet connectivity, dense populations, unequal access to water, and limited social safety nets. COVID-19 is challenging the public health and socio-political systems of all affected African countries. The COVID-19 Pandemic has enhanced indications of how dire the consequences of a highly infectious disease Pandemic could be. In line with the steps being taken across the globe to control and contain COVID-19 Pandemic, African countries are preparing for the worst effects of this pandemic and ensuing deep recession. The spread of the pandemic will eventually stop, and the international system will find a balance, but most of the damage will be felt particularly by African countries.
